# Quetiapine Attenuates Schizophrenia-Like Behaviors and Demyelination in a MK-801–Induced Mouse Model of Schizophrenia

**DOI:** 10.3389/fpsyt.2020.00843

**Published:** 2020-08-19

**Authors:** Jue He, Qian Zu, Chunyan Wen, Qianqian Liu, Pan You, Xinmin Li, Wenqiang Wang

**Affiliations:** ^1^ Department of Mental Health Research, Xiamen Xian Yue Hospital, Xiamen, China; ^2^ Institute of Neurological Disease, First Affiliated Hospital, Henan University, Kaifeng, China; ^3^ Department of Psychiatry, Faculty of Medicine and Dentistry, University of Alberta, Edmonton, AB, Canada

**Keywords:** quetiapine, MK-801, schizophrenia, memory, prepulse inhibition deficit, demyelination, brain-derived neurotrophic factor

## Abstract

Brain demyelination is possibly one of the main pathological factors involved in schizophrenia, and targeting on myelination may be a useful strategy for schizophrenia treatment. Quetiapine, a widely used atypical antipsychotic drug for schizophrenia treatment, has been reported to have neuroprotective effects on cerebral myelination in a demyelination animal model. The objective of the present study was to evaluate the effect and underlying neuroprotective mechanism of quetiapine on the schizophrenia-like behaviors and possible cerebral demyelination induced by MK-801, an N-methyl-D-aspartate glutamate receptor antagonist. Mice were treated with chronic quetiapine (10 mg/kg/day, intraperitoneally) for 28 days. From day 22 to 28, 1 h after the administration of quetiapine, the mice were administered MK-801 (2 mg/kg/day, subcutaneously). The positive symptom of schizophrenia was measured in a locomotor activity test on day 29, the memory was evaluated by a Y-maze test on day 30, and the sensorimotor gating deficit in mice was measured by prepulse inhibition test on day 31. After the behavioral tests, the protein expression of myelin basic protein (MBP) was measured by Western Blot, and the protein expression of brain-derived neurotrophic factor (BDNF) was measured by ELISA in the frontal cortex of mice. Our results showed quetiapine attenuated schizophrenia-like behaviors including hyperactivity, memory impairment, and sensorimotor gating deficit in the MK-801 mice. In the same time, quetiapine attenuated demyelination, concurrent with attenuated BDNF decrease in the brain of MK-801-injected mice. These results suggest that the beneficial effects of quetiapine on schizophrenia might be partly related to its neuroprotective effect on brain myelin basic protein and its upregulating neuroprotective proteins such as BDNF, and indicate that modulation of cerebral demyelination could be a novel treatment target of schizophrenia.

## Introduction

Although the etiology and pathogenesis of schizophrenia remain poorly understood, chemical and physiologic imaging supports there is dysfunction of neural networks in the brains of schizophrenic patients (1). Myelin dysfunction produces abnormal connectivity of neural networks. An abnormality of brain myelin affecting white matter may be involving the pathological process underlying schizophrenia (2–4). Abnormalities of myelination neuro-imaging and ultrastructural pathology of myelinated fibers and oligodendrocytes have been observed in schizophrenic brains (5, 6). There are dysregulation of myelination-related genes revealed by genome-wide expression analysis, and myelin-associated mRNA and protein expression deficits in the brains of schizophrenia patients (7, 8). Oligodendrocytes are the myelin-producing cells in the central nervous system. Oligodendrocyte progenitor cells contain functional N-methyl-D-aspartate (NMDA) receptors (9), and NMDA glutamate receptors mediate Ca2+ accumulation in central myelin in response to chemical ischemia *in vitro* (10). Our previous study has shown phencyclidine, an NMDA receptor antagonist, induced brain demyelination when administrated in postnatal rats (11). MK-801, another NMDA receptor antagonist, can cause schizophrenia-like behaviors including prepulse inhibition (PPI) deficit and memory impairment in animals, and is used as a pharmacological model of schizophrenia (12, 13).

Quetiapine, an atypical antipsychotic drug, has been widely used in the treatment of schizophrenia and has shown its efficacy in treating positive and negative symptoms, as well as cognitive impairment in schizophrenia patients (14–16). In animal studies, quetiapine enhanced oligodendrocyte regeneration and myelin-repair in cuprizone-induced demyelination of mouse brain (17), and prevented oligodendrocyte and myelin losses in the hippocampus of global cerebral ischemia mice (18). The beneficial effects of quetiapine on cerebral oligodendrocyte and demyelination may be related to its neuroprotective effects. Previous studies have shown that quetiapine attenuated the immobilization stress-induced decrease in expression of brain-derived neurotrophic factor (BDNF, a neuroprotective protein) (19), promoted neuroplasticity *via* the up-regulation of BDNF (20) in rat hippocampus, and modulated conditioned anxiety by affecting brain BDNF expression in Alzheimer’s transgenic mice (21). BDNF is relevant for schizophrenia-related phenotypes, and disruption of BDNF signaling is associated with altered synaptic plasticity and neurodevelopment of schizophrenia (22). Cerebral myelination can be evaluated by measuring the expression of myelin basic protein (MBP) (11). Both cerebral myelination and BDNF play important roles in the pathology and neurodevelopment of schizophrenia (5, 6, 22). BDNF is a neuroprotective protein, while neuroprotective factors could be beneficial on cerebral myelin damage.

In order to evaluate whether modulation of cerebral demyelination could be a novel treatment target of schizophrenia, chronic administration of MK-801 was used to induce schizophrenia-like behaviors and possible cerebral demyelination by measuring MBP, and the effect and BDNF-related neuroprotective mechanism of quetiapine on the MK-801–induced schizophrenia-like behaviors and demyelination was investigated in mice in the present study.

## Materials and Methods

### Animals

All procedures with animals were performed in accordance with the Chinese Council on Animal Welfare and Ethics Guidelines (GB/T35892-2018) and approved by the Care and Use of Laboratory Animal Ethics Committee of Xiamen Xian Yue Hospital. In total 40 female ICR (CD1) mice (Xiamen University Laboratory Animal Center, Xiamen, Fujian Province, China) of 6 weeks old at the beginning of the experiments were used. Mice were housed five per cage, with free access to food and water under controlled laboratory conditions (a 12:12 hour light/dark cycle with room temperature 20°C ± 1°C). All behavioral tests were performed in the light phase.

### Drug Treatment

MK-801 [(+)-MK-801 hydrogen maleate] purchased from Sigma-Aldrich (St. Louis, Missouri, USA) and quetiapine obtained from AstraZeneca Pharmaceuticals (Macclesfield, UK) were both freshly dissolved in saline. Mice were treated with chronic quetiapine (0 or 10 mg/kg/day, intraperitoneally) for 28 days. From day 22 to 28, 1 h after the administration of quetiapine, the mice were administered MK-801 (0 or 2 mg/kg/day, subcutaneously). No mortality was observed in the MK-801-treated mice. The volume of all injected solutions was 10 ml/kg. Totally 40 mice were randomly assigned into four groups (n=10 in each group): saline + saline (CON), Quetiapine + saline (Que), MK-801 + saline (MK), MK-801 + Quetiapine (MK+Que).

### Behavioral Testing

#### Locomotion Measurement

On day 29, locomotor activity was measured for 60 min in a plexiglas activity box (40 cm × 40 cm × 22 cm high) surrounded with an activity meter (Opto-Varimex Mini; Columbus Instruments, Columbus, OH) (11). The locomotor activity at the first 10 min out of 60 min in the locomotor activity test was also measured. Locomotor activity included central and peripheral activity measured by the activity meter. There is a 12 × 12 photobeam array located 1 cm above the box floor, and photobeam interruptions were recorded by the activity meter. An interruption of a light beam was recorded as an activity count. The apparatus was located in a sound-attenuated room and illuminated with a 40 W light source at the top. Locomotor activities were used to evaluate “positive-like symptom” of schizophrenia. Impairment in locomotor activities has been observed in several demyelination animal models (23, 24).

#### Y-Maze Test

On day 30, spatial working memory performance in mice was assessed by recording spontaneous alternation behavior in a Y-maze (25). The maze was made of black painted wood. Each arm of the Y-maze was 40-cm long, 12-cm high, 3-cm wide at the bottom and 10-cm wide at the top, and positioned at an equal angle. The mice were individually placed at the end of one arm and allowed to move freely through the maze during an 8-min session. Mice tend to explore the maze systematically, entering each arm in turn. The ability to alternate requires that the mice know which arm they have already been visited. The sequence of arm entries was recorded visually. Spontaneous alternation behavior was defined as the entry into all three arms on consecutive choices in overlapping triplet sets. The percent spontaneous alternation behavior was calculated as the ratio of actual to possible alternations (defined as the total number of arm entries minus 2) multiplied by 100 (26).

#### PPI of the Startle Rsponse Test

On day 31, the sensorimotor gating in mice was evaluated in a PPI test (27). PPI of the startle response provides an operational measure of sensorimotor gating, a putative neural mechanism that inhibits the processing of extraneous sensory, cognitive and motor information (28). PPI refers to the normal reduction in startle magnitude that occurs when an abrupt startling stimulus is preceded 30 to 500 ms by a weak prestimulus (28). Disruption of PPI has been well documented in schizophrenia patients (29, 30).

Each mouse was placed into a small Plexiglas cylinder connecting motion sensors within a large sound-attenuating chamber (San Diego Instruments, San Diego, CA). The motion sensors recorded voltage changes in arbitrary units (mV) that were digitized and stored by the computer controlling the delivery of acoustic stimuli through loudspeakers mounted 21 cm above the plastic cylinders. Motion sensors were calibrated daily with the SR-LAB Standardization Unit (San Diego Instruments, San Diego, CA). The background sound level (70 dB) and calibration of the acoustic stimuli was confirmed daily with a digital sound level meter. Startle pulse was set to 120 dB and prepulse intensities were set to 3 dB (PP3), 6 dB (PP6) and 12 dB (PP12) above background noise level. The test session consisted of 64 trials and included five trial types (startle pulse-alone, PP3, PP6, PP12, and no-stimulus) in four blocks. Blocks 1 and 4 included six startle pulse-alone trials in each block, and blocks 2 and 3 included six startle pulse-alone trials, five PP3, five PP6, five PP12, and five no-stimulus in each block. Duration of acoustic stimuli was set to 20 ms for prepulses and 40 ms for startle pulses, and interstimulus interval between prepulse and startle pulse was set to 100 ms (from onset to onset).

After a 5-min habituation period, PPI-test sessions were conducted. The intertrial intervals with an average of 15 s were varied from 5 to 23 s in pseudo-random order in the session, and a total PPI test time for each mouse was approximately 23 min. The maximum response in 65-ms recording window was used as the startle amplitude for each trial. Levels of PPI at each prepulse sound level were calculated in blocks 2 and 3 as (1-averaged response amplitude in trials with a prepulse stimulus and startle stimulus/averaged response amplitude in trials with the startle stimulus alone) x 100%. The averaged response amplitude in trials with the startle stimulus alone and the averaged response amplitude in trials with no-stimulus in blocks 2 and 3 were also calculated.

### Western Blot Analysis

One day after the PPI test on Day 32, the mice (five mice in each group) were sacrificed and perfused with 0.1 M phosphate-buffered saline (PBS, pH 7.4), and the frontal cortex from right hemisphere was dissected, frozen in dry-ice powder and stored at −70°C until used (31). The frontal cortex samples from each mouse were homogenized at 4°C in a lysis buffer [50 mM Tris-HCl, 150 mM NaCl, 10 mM EDTA, 10 mM NaF, 1 mM sodium orthovanadate, 1% NP-40, 10 mM sodium pyrophosphate decahydrate, 0.5 mM DTT, 0.2 mM PMSF, and Complete Mini Protease Inhibitor Cocktail (Roche Diagnostics, Laval, Qc, Canada); pH 7.4]. The lysates were centrifuged twice at 10,000*g* for 10 min. The protein concentration of the supernatant was determined using a BCA protein assay kit (Pierce, Rockford, IL, USA). An aliquot of each sample containing equal amounts of total protein (50 μg/8–9 μl) was denatured in a protein loading buffer, separated on a 12% polyacylamide gel, and subsequently transferred to a polyvinylidene difluoride membrane (Biorad, Hercules, CA, USA). After being blocked by 5% skim milk powder in TBST (10 mM Tris-HCl, 150 mM NaCl, 0.05% Tween 20; pH 7.4), the membranes were incubated overnight at 4°C with an anti-MBP (18 kDa, 1:1,000, mouse monoclonal; sc-271524, Santa Cruz Biotechnology, Santa Cruz, CA, USA) antibody or anti–β-actin (42 kDa, 1:5,000, mouse monoclonal; A1978, Sigma-Aldrich, St. Louis, MO, USA) antibody. The membranes were then washed with TBST and probed with horseradish peroxidase-conjugated anti-mouse secondary antibody for 2 h at room temperature. The immune complexes were detected by an ECL chemiluminescence system (Amersham, Buckinghamshire, UK) and exposed to high performance chemiluminescence film (Amersham, Buckinghamshire, UK). The MBP or β-actin band in the film was scanned using Vista Scan software. Then the band intensities (optical density) of MBP or β-actin were analyzed by densitometry (density × area) using an Image-Pro Plus image analysis system (Media Cybernetics, Silver Spring, MD, USA), and the MBP/β-actin ratio was calculated. The results of MBP/β-actin ratio in each group were standardized to the measurement results of the CON group (as 100%).

### ELISA Measurement

One day after the PPI test on Day 32, the mice (5 mice in each group) were sacrificed and perfused with 0.1 M phosphate-buffered saline (PBS, pH 7.4). The frontal cortex from right hemisphere was dissected on the back surface of an iced tissue culture dish covered by a PBS-wetted filter paper, and was kept for BDNF ELISA measurement. The level of BDNF was quantified using a commercial Mouse BDNF ELISA kit (Fitzgerald Industries International, North Acton, MA) according to the manufacturer’s protocol. All measurements were done in duplicate. The level of BDNF was corrected for total protein of brain tissue and dilution factor, and was standardized to that in the CON group (as 100%).

### Statistical Analysis

All results were expressed as means ± S.E.M. The significance of differences was determined by two-way ANOVA, followed by a Newman-Keuls *post hoc* test for multiple comparisons. A two-tailed *t*-test for independent samples was used for two-group comparisons. A *P* value of less than 0.05 was regarded as statistically significant.

## Results

### Quetiapine Attenuated Schizophrenia-Like Behaviors in the MK-801 Mice

#### Quetiapine Attenuated Hyperlocomotor Activity in the MK-801 Mice

**Figure 1:**
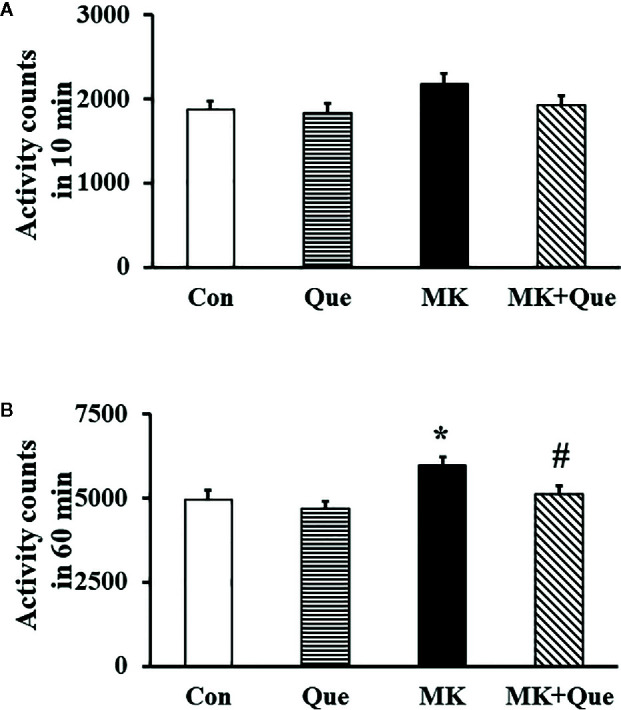
Locomotor activity in the mice of control (Con), quetiapine (Que), MK-801 (MK), and MK-801+quetiapine (MK+Que) groups. Locomotor activity was measured at the first 10 min **(A)** of 60 min and at the 60 min **(B)**. Quetiapine significantly attenuated the hyperlocomotor activity measured at 60 min in the MK-801 mice. n=10 in each group. Mice were treated with chronic quetiapine (10 mg/kg/day, intraperitoneally) for 28 days. From day 22 to 28, 1 h after the administration of quetiapine, the mice were administered MK-801 (2 mg/kg/day, subcutaneously). On day 29, locomotor activity was measured for 60 min in a plexiglas activity box. Results are expressed as means ± S.E.M. *P < 0.05 vs. Con, ^#^ *P *< 0.05 vs. MK.

To evaluate schizophrenia-like behavior, a locomotor activity test was performed to investigate locomotion in mice. As shown in **Figure 1**, although there was no significant difference in the total number of locomotor activity counts in the first 10 min among the Con, Que, MK and MK+Que groups (**Figure 1A**), two-way ANOVA showed that MK-801 and quetiapine produced a significant change in the total number of locomotor activity counts in 60 min in mice (**Figure 1B**) [*F*(MK-801)(1,36)=8.39, *P*<0.01; *F*(quetiapine)(1,36)=4.54, *P*<0.05], and that there was no interaction between MK-801 and quetiapine. The total number of locomotor activity counts in the MK-801 mice was higher than that in the control mice, and quetiapine significantly attenuated hyperlocomotor activity in the MK-801 mice in the test of 60 min (*t*-test, **Figure 1B**).

#### Quetiapine Prevented the Spatial Working Memory Impairment in the MK-801 Mice in a Y-Maze Test

**Figure 2:**
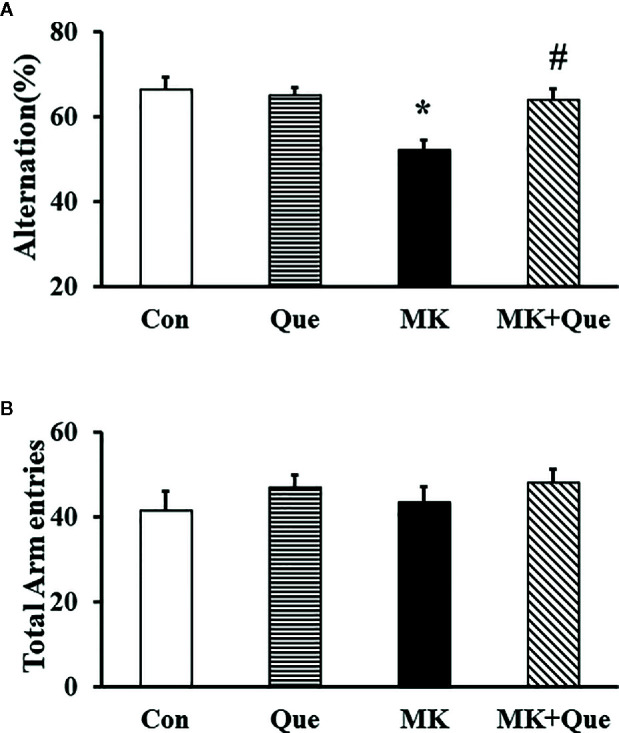
Spatial working memory in the mice of control (Con), quetiapine (Que), MK-801 (MK), and MK-801+quetiapine (MK+Que) groups. Quetiapine attenuated the decrease of spontaneous alternation behavior in the MK-801 mice **(A)**, and quetiapine and MK-801 had no effect on the number of total arm entries **(B)** during the Y-maze test in mice. n=10 in each group. Mice were treated with chronic quetiapine (10 mg/kg/day, intraperitoneally) for 28 days. From day 22 to 28, 1 h after the administration of quetiapine, the mice were administered MK-801 (2 mg/kg/day, subcutaneously). On day 30, spatial working memory performance in mice was assessed by recording spontaneous alternation behavior in a Y-maze. Results are expressed as means ± S.E.M. *P < 0.05 vs. Con, ^#^ *P *< 0.05 vs. MK.

Spatial working memory performance in mice was assessed by recording spontaneous alternation behavior in a Y-maze test (25). In this test, mice tend to explore the maze systematically, entering each arm in turn. The ability to alternate requires that the mice remember which arm they have just visited. As shown in **Figure 2A**, two-way ANOVA showed that MK-801 and quetiapine produced a significant change in the alternation performance in mice [*F*(MK-801)(1,36)=9.42, *P*<0.01; *F*(quetiapine)(1,36)=4.36, *P*<0.05], and that there was an interaction between the two factors [*F*(MK-801 × quetiapine)(1,36)=7.14, *P*<0.05]. A *post hoc* analysis revealed that the alternation performance in the MK-801 mice was less than that in the control mice, and that quetiapine significantly prevented the decrease of the alternation performance in the MK-801 mice (**Figure 2A**). There was no difference in the number of total arm entries among the Con, Que, MK and MK+Que groups (**Figure 2B**).

#### Quetiapine Attenuated Sensorimotor Gating Deficit in the MK-801 Mice in a PPI Test

**Figure 3:**
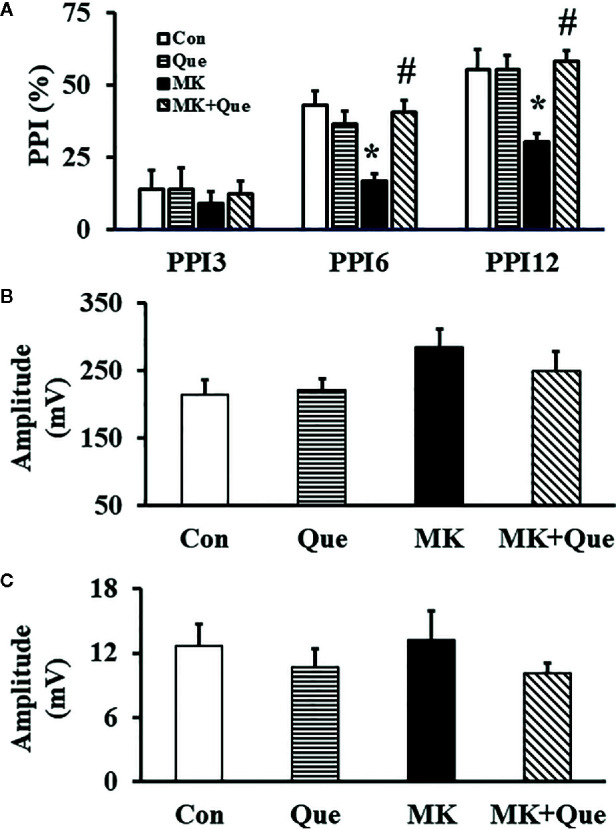
prepulse inhibition (PPI) **(A)**, auditory startle respone to the startle stimulation **(B)** and auditory response of the no-stimulus **(C)** in a PPI test in the mice of control (Con), quetiapine (Que), MK-801 (MK), and MK-801+quetiapine (MK+Que) groups. Quetiapine prevented the decrease of the PPI (%) in the MK-801 mice at PPI6 and PPI12, and quetiapine and MK-801 had no effects on the PPI (%) at PPI3, on the auditory startle respone to the startle stimulation and on the auditory response of the no-stimulus. n=10 in each group. Mice were treated with chronic quetiapine (10 mg/kg/day, intraperitoneally) for 28 days. From day 22 to 28, 1 h after the administration of quetiapine, the mice were administered MK-801 (2 mg/kg/day, subcutaneously). On day 31, the sensorimotor gating in mice was evaluated in a PPI test. Results are expressed as means ± S.E.M. *P < 0.05 vs. Con, ^#^ *P *< 0.05 vs. MK.

To investigate the sensorimotor gating in mice after MK-801 and quetiapine treatment, a PPI test was performed. As shown in **Figure 3A**, two-way ANOVA showed that MK-801 and quetiapine produced a significant change in the PPI (%) at PPI6 [*F*(MK-801)(1,36)=7.32, *P*<0.05; *F*(quetiapine)(1,36)=4.57, *P*<0.05] and PPI12 [*F*(MK-801)(1,36)=5.42, *P*<0.05; *F*(quetiapine)(1,36)=8.52, *P*<0.01] in mice, and that there was an interaction between the two factors in the PPI (%) at PPI6 [*F*(MK-801 × quetiapine)(1,36)=14.12, *P*<0.001] and PPI12 [*F*(MK-801 × quetiapine)(1,36)=8.62, *P*<0.01]. A *post hoc* analysis revealed that the PPI (%) in the MK-801 mice was less than that in the control mice, and that quetiapine significantly prevented the decrease of the PPI (%) in the MK-801 mice at PPI6 and PPI12 (**Figure 3A**). There was no difference in the PPI (%) at PPI3 among the Con, Que, MK and MK+Que groups (**Figure 3A**).

The auditory startle response to the startle stimulus alone was measured in the PPI section in mice. As shown in **Figure 3B**, there was no significant difference in the auditory startle response to the startle stimulus in the PPI section among the Con, Que, MK and MK+Que groups.

In order to evaluate the basal motor activity in the cylinder during the PPI test, mouse movements were evaluated by the no-stimulus trials without any stimulation. As shown in **Figure 3C**, there was no significant difference in the amplitude of response for no-stimulus in the PPI section among the Con, Que, MK and MK+Que groups.

### Quetiapine Prevented the Decrease of MBP Expression in the Frontal Cortex of MK-801 Mice

**Figure 4:**
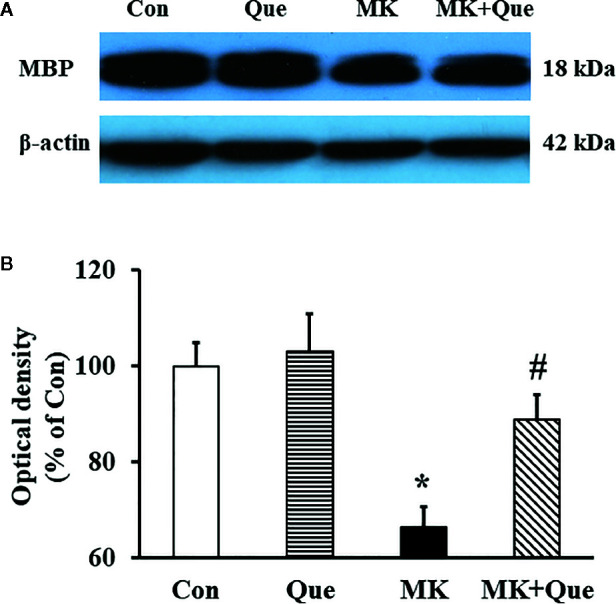
**(A)** Representative western blot bands of myelin basic protein (MBP) protein expression in the frontal cortex of mice in control (Con), quetiapine (Que), MK-801 (MK), and MK-801+quetiapine (MK+Que) groups. **(B)** Histogram showing the quantification of the immmunochemically reactive bands in the Western blot of MBP in the frontal cortex of mice in the Con, Que, MK and MK+Que groups. Quetiapine attenuated the decrease of MBP level in the frontal cortex of MK-801 mice. n=5 in each group. Mice were treated with chronic quetiapine (10 mg/kg/day, intraperitoneally) for 28 days. From day 22 to 28, 1 h after the administration of quetiapine, the mice were administered MK-801 (2 mg/kg/day, subcutaneously). One day after behavioral testing, the mice were sacrificed for MBP protein analysis by western blot. Results are expressed as means ± S.E.M. *P < 0.05 vs. Con, ^#^ *P* < 0.05 vs. MK.

MBP is a protein believed to be important in the process of myelination of nerves in the central nervous system, and cerebral myelination was evaluated by the expression of MBP using Western blot in mice (31, 32). Representative western blot bands of MBP in the mice of Con, Que, MK and MK+Que groups are shown in **Figure 4A**. The optical densities of Western blot bands were quantified. As shown in **Figure 4B**, two-way ANOVA showed that MK-801 and quetiapine produced a significant change in the expression of MBP in the frontal cortex of mice [*F*(MK-801)(1,16)=17.56, *P*<0.001; *F*(quetiapine)(1,16)=5.04, *P*<0.05], and that there was no interaction between MK-801 and quetiapine. The expression of MBP in the frontal cortex of MK-801 mice was less than that of control mice, and quetiapine significantly prevented the decrease of the expression of MBP in the frontal cortex of MK-801 mice (*t*-test, **Figure 4B**).

### Quetiapine Attenuated the Decrease of BDNF Level in the Frontal Cortex of MK-801 Mice

**Figure 5:**
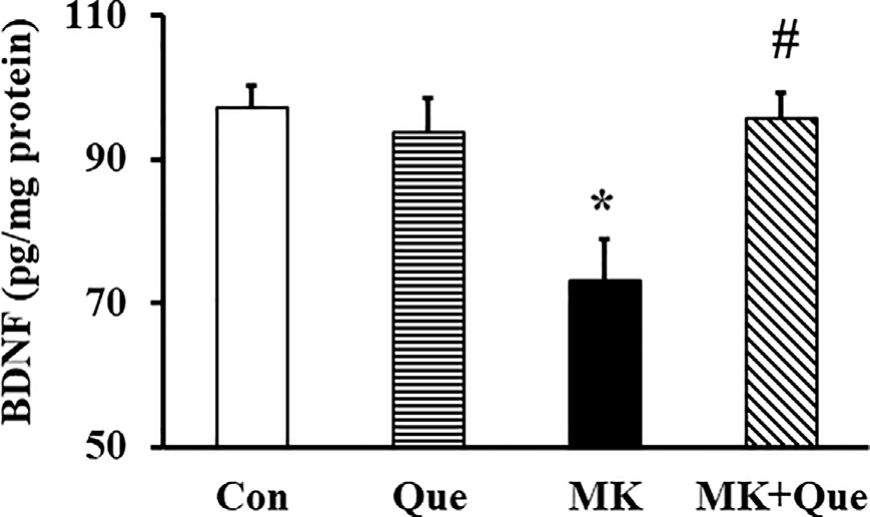
Brain-derived neurotrophic factor (BDNF) protein level in the frontal cortex of mice in control (Con), quetiapine (Que), MK-801 (MK), and MK-801+quetiapine (MK+Que) groups. Quetiapine attenuated the decrease of BDNF level in the frontal cortex of MK-801 mice. n=5 in each group. Mice were treated with chronic quetiapine (10 mg/kg/day, intraperitoneally) for 28 days. From day 22 to 28, 1 h after the administration of quetiapine, the mice were administered MK-801 (2 mg/kg/day, subcutaneously). One day after behavioral testing, the mice were sacrificed for BDNF protein analysis by ELISA. Results are expressed as means ± S.E.M. *P < 0.05 vs. Con, ^#^ *P *< 0.05 vs. MK.

To investigate the neuroprotective mechanism of quetiapine involving in its beneficial effects on schizophrenia-like behaviors and demyelination in the MK-801 mice, the BDNF level in the frontal cortex of mice was measured by ELISA. As shown in **Figure 5**, two-way ANOVA showed that MK-801 and quetiapine produced a significant change in the BDNF level in the frontal cortex of mice [*F*(MK-801)(1,16)=6.00, *P*<0.05; *F*(quetiapine)(1,16)=4.50, *P*<0.05], and that there was an interaction between the two factors [*F*(MK-801 × quetiapine)(1,16)=8.57, *P*<0.01]. A *post hoc* analysis revealed that the BDNF level in the frontal cortex of the MK-801 mice was less than that in the control mice, and that quetiapine significantly attenuated the BDNF level in the frontal cortex of the MK-801 mice (**Figure 5**).

## Discussion

The pathological process underlying schizophrenia may involve an abnormality of brain myelin affecting white matter (2–4). The myelin dysfunction hypothesis of schizophrenia was first tested by an atypical antipsychotic, quetiapine, in a schizophrenia animal model induced by MK-801 in the present study. Our study has shown that quetiapine attenuated the schizophrenia-like behaviors and cerebral demyelination in the MK-801–injected mice.

To elucidate the etiology and pathogenesis of schizophrenia, NMDA receptor antagonists including MK-801 and phencyclidine have been used to induce pharmacological animal models of schizophrenia (33). Consistent with previous studies that presented schizophrenia-like behaviors in the MK-801 injected animals (12, 13, 34), MK-801 administrated mice demonstrated the increase of locomotor activity, spatial working memory impairment and sensorimotor gating deficit. Quetiapine, an atypical antipsychotic drug, has been widely used in the treatment of schizophrenia and has shown its efficacy in treating positive and negative symptoms, as well as cognitive impairment in schizophrenia patients (14–16). In the present study, quetiapine attenuated schizophrenia-like behaviors including hyperlocomotor activity, spatial working memory impairment, and sensorimotor gating deficit in the MK-801 mice.

Although the pathogenesis of schizophrenia remains unknown, abnormalities of myelination neuro-imaging and ultrastructural pathology of myelinated fibers and oligodendrocytes have been observed in schizophrenic brains (5, 6). Oligodendrocytes are the myelin-producing cells in the central nervous system. Oligodendrocyte progenitor cells contain functional N-methyl-D-aspartate (NMDA) receptors (9). Our previous study has shown phencyclidine, an NMDA receptor antagonist, induced brain demyelination when administrated in postnatal rats (11). In the present study, MK-801 decreased the protein expression of MBP which believed to be important in the process of myelination of nerves in the central nervous system in the frontal cortex of mice (32). A study by using unbiased stereological methods and a transmission electron microscope technique has demonstrated MK-801 induced splitting lamellae of myelin sheaths and segmental demyelination in the corpus callosum of mice (35).

Quetiapine attenuated the schizophrenia-like behaviors induced by MK-801, a NMDA receptor antagonist, concurrent with attenuated demyelination in the frontal cortex of MK-801 mice. Oligodendrocyte progenitor cells contain functional NMDA receptors, and NMDA receptor antagonists could induce brain demyelination (9, 11). Previous studies have demonstrated that quetiapine enhanced oligodendrocyte regeneration and myelin-repair in cuprizone-induced demyelination of mouse brain (17), and prevented oligodendrocyte and myelin losses in the hippocampus of global cerebral ischemia mice (18). Therefore, quetiapine and MK-801 may target on both oligodendrocyte progenitor cells and mature oligodendrocytes to affect substantial myelination in the current study, and further studies are needed to be done to support this description. The demyelination lesions in frontal and parietal brain regions correlate particularly well with the presence of psychotic symptoms (36). The beneficial effects of quetiapine on the MK-801–induced schizophrenia-like behaviors and cerebral demyelination indicate that schizophrenia-like behaviors in the MK-801–injected mouse may be a consequence of a myelination deficit in the frontal cortex, and support that the pathological process underlying schizophrenia involves an abnormality of CNS myelin affecting white matter. In addition, since dopamine receptors are very important factors involving in schizophrenia, further studies are necessary to elucidate whether dopamine D1 or D2 receptor is involved in the effect of quetiapine on myelination in the MK-801 mouse model.

The neurotrophic and neuroprotective effect of quetiapine may contribute to its attenuating effects on cerebral demyelination and schizophrenia-like behaviors in the MK-801 mice. BDNF, an important neurotrophin, mainly expressed and distributed in brain, has neuroprotective effects. BDNF may act on oligodendrocyte expressed tropomyosin-related kinase (Trk) B to potentiate and enhance myelination (37), and is relevant for schizophrenia-related phenotypes (22). Therefore, BDNF may be one of causes of myelin repair in the present study. Quetiapine attenuated the immobilization stress-induced decrease of BDNF in rat hippocampus (19). In an Alzheimer’s disease transgenic model, quetiapine attenuated the decrease in number of BDNF positive cells in basolateral amygdala and hippocampus of Alzheimer’s disease mice (21). Therefore, quetiapine may benefit the MK-801–induced schizophrenia-like behaviors and reserve cerebral demyelination by its potential neuroprotective effects on oligodendrocyte and myelin. However, further studies are necessary to elucidate the role of BDNF in oligodendrocytes in the therapeutic effect of quetiapine in the MK-801–induced schizophrenia animal model. In addition, the limitation of our current study is lacking direct morphological evidence showing the effects of quetiapine and MK-801 on oligodendrocytes and myelin structure, and we will extend our study to analyze staining changes of mature oligodendrocytes, oligodendrocyte progenitor cells and myelin sheaths in the cortex of the quetiapine treated MK-801 mice in the future.

The present study suggests that the beneficial effects of quetiapine on schizophrenia might be related to its neuroprotective effect on brain myelin basic protein and its upregulating neuroprotective proteins such as BDNF, and indicate that modulation of cerebral demyelination could be a novel treatment target of schizophrenia.

## Data Availability Statement

The datasets generated for this study are available on request to the corresponding authors.

## Ethics Statement

The animal study was reviewed and approved by Care and Use of Laboratory Animal Ethics Committee of Xiamen Xian Yue Hospital.

## Author Contributions 

JH and WW designed the research with the contribution of all authors. QZ and CW performed behavioral experiments. QL and PY performed experiments of Western Blot and ELISA. JH and XL wrote the manuscript with comments and contributions from all authors.

## Funding 

This work was supported by grants from National Nature Science Foundation of China (81871058, 81671266), Fujian Provincial Department of Science and Technology (2017D0007), and Huanghe Scholar Foundation of Henan University for JH.

## Conflict of Interest

The authors declare that the research was conducted in the absence of any commercial or financial relationships that could be construed as a potential conflict of interest.
